# Is there still a place for retroperitoneal lymph node dissection in clinical stage 1 nonseminomatous testicular germ-cell tumours? A retrospective clinical study

**DOI:** 10.1186/s12894-018-0412-x

**Published:** 2018-10-26

**Authors:** K.-P. Dieckmann, P. Anheuser, M. Kulejewski, R. Gehrckens, B. Feyerabend

**Affiliations:** 1 Albertinen-Krankenhaus Hamburg, Klinik für Urologie, Hamburg, Germany; 2 Asklepios Klinik Altona, Urologische Abteilung, Hodentumorzentrum Hamburg, Hamburg, Germany; 3 Albertinen-Krankenhaus Hamburg, Klinik für Diagnostische Radiologie, Hamburg, Germany; 4 MVZ Hanse Histologikum, Hamburg, Germany; 5 Asklepios Klinik Altona, Hodentumorzentrum Hamburg, Paul Ehrlich Strasse 1, 22763 Hamburg, Germany

**Keywords:** Testicular germ cell tumour, Nonseminomatous tumour, Lymph node dissection, Teratoma

## Abstract

**Background:**

Primary retroperitoneal lymph node dissection (RPLND) ultimately lost its role as the standard management of clinical stage (CS) 1 nonseminomatous (NS) testicular germ cell tumours (GCTs) in Europe when the European Germ Cell Cancer Consensus Group released their recommendations in 2008. Current guide-lines recommend surgery only for selected patients but reasons for selection remain rather ill-defined. We evaluated the practice patterns of the management of CS1 patients and looked specifically to the role of RPLND among other standard treatment options.

**Methods:**

We retrospectively evaluated the treatment modalities of 75 consecutive patients treated for CS1 NS at one centre during 2008–2017. The patients undergoing RPLND were selected for a closer review. Particular reasons for surgery, clinical features of patients, and therapeutic outcome were analyzed using descriptive statistical methods.

**Results:**

Twelve patients (16%) underwent nerve-sparing RPLND, nine surveillance, 54 had various regimens of adjuvant chemotherapy. Particular reasons for surgery involved illnesses precluding chemotherapy (*n* = 2), patients´ choice (*n* = 4), and teratomatous histology of the primary associated with equivocal radiologic findings (*n* = 6). Five patients had lymph node metastases, two received additional chemotherapy. Antegrade ejaculation was preserved in all cases. One patient had a grade 2 complication that was managed conservatively. All RPLND-patients remained disease-free.

**Conclusions:**

Primary RPLND is a useful option in distinct CS1 patients, notably those with concurrent health problems precluding chemotherapy, and those with high proportions of teratoma in the primary associated with equivocal radiological findings. Informed patient’s preference represents another acceptable reason for the procedure. RPLND properly suits the needs of well-selected patients with CS1 nonseminoma and deserves consideration upon clinical decision-making.

## Background

Patients with clinical stage (CS) 1 nonseminomatous (NS) testicular germ cell tumors (GCTs) can be successfully managed with quite different treatment methods [1]. Retroperitoneal lymph node dissection (RPLND) used to be the standard of care for a fifty years period from the end of world war II [2, 3] to the late nineties of the last century [4, 5]. In European countries, it was then gradually replaced by surveillance strategies with chemotherapy to be applied at the time of progression [6–9]. Primary prophylactic chemotherapy with two cycles of the cisplatin-etoposide-bleomycin (PEB) regimen came into use as another alternative around the turn of the century [10]. Currently, a risk-adapted strategy using vascular invasion (VI) of the primary tumour as a risk indictor [11] is the most preferred option with surveillance in the absence of the risk factor and prophylactic chemotherapy with one cycle of PEB if vascular invasion is detected in the primary [12]. In 2008, the European Germ Cell Cancer Consensus Group (EGCCCG) released guide-lines that virtually abandoned RPLND as the standard of management of CS1 NS in European countries [13]. Since that time patients underwent stratifying with regard to the presence of risk factors for progression. If vascular invasion of the primary was present, adjuvant chemotherapy became the standard way of care while surveillance and RPLND were considered merely as options for rare and specific cases. In patients without risk factor, surveillance was considered the standard way of treatment assigning RPLND only a role for exceptional circumstances. In the most recent guide-line of the European Association of Urology (EAU), surveillance is considered one standard option for all patients with nonseminoma CS1 while risk-adapted strategy is regarded another equally effective standard option [14]. RPLND is justified only in the few cases when “conditions are against surveillance and chemotherapy”. Unfortunately, no further definitions were given to clarify those “conditions” and thus, decision-making was left to care-givers and patients. Currently, the degree of utilization of RPLND in European countries is largely unknown [15, 16]. The aim of the present study is to evaluate the patterns of care applied to NS CS1 patients in a testicular cancer unit in Northern Germany and to specifically look to the utilization of RPLND.

## Patients, methods

**Fig. 1:**
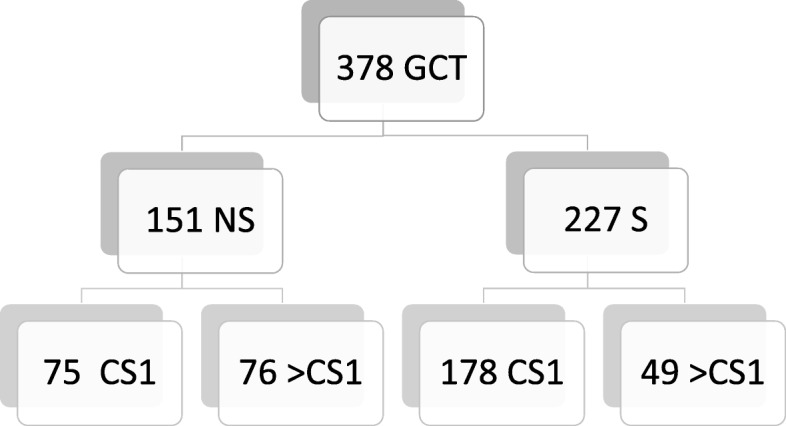
Histology and clinical stages in 378 patients with testicular germ cell tumours treated in a single institution, 2008–2017 (numbers of patients). GCT germ cell tumours; S seminoma; NS nonseminoma; CS clinical stage

**Fig. 2:**
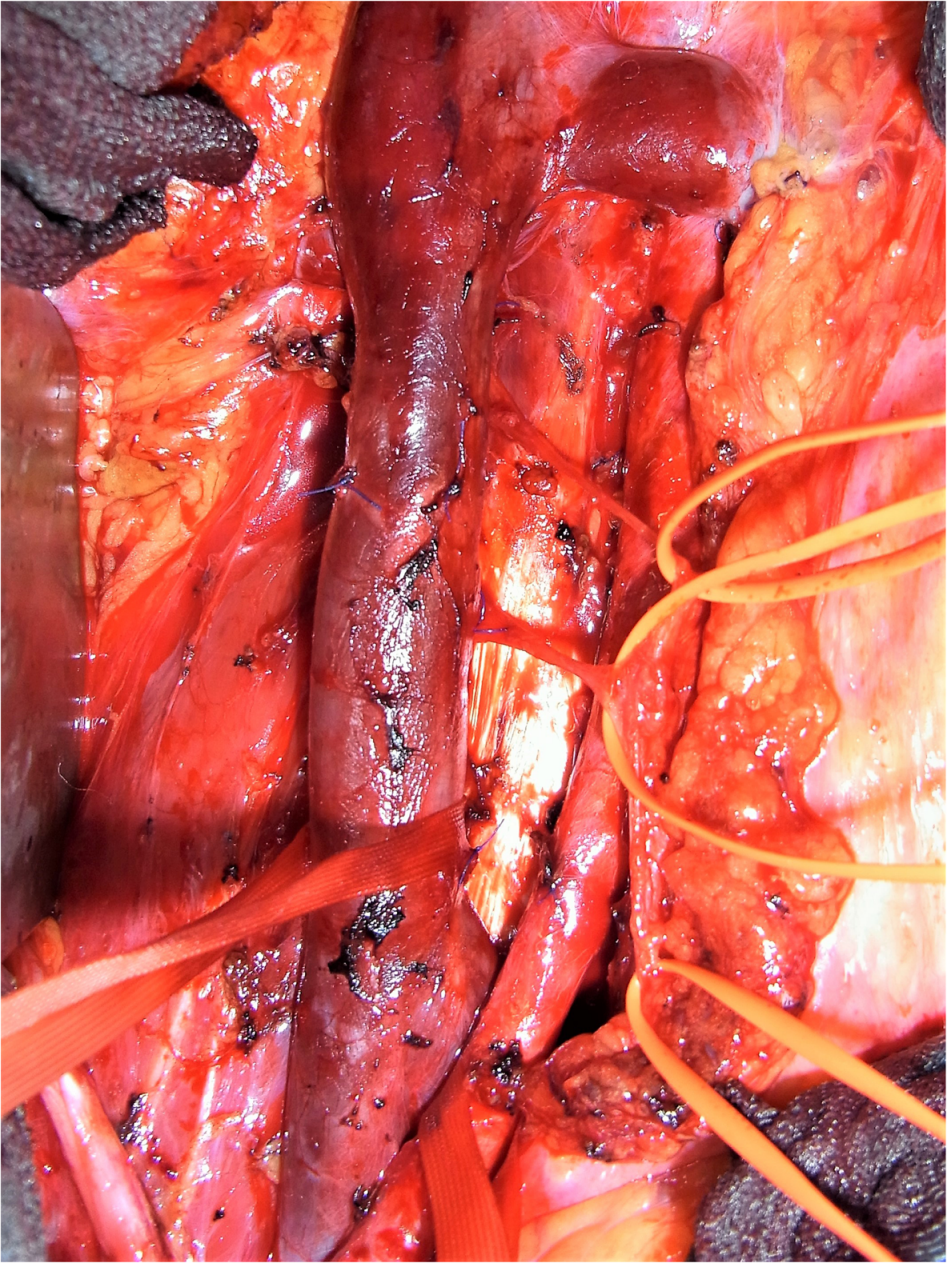
Intraoperative site during right sided nerve sparing retroperitoneal lymph node dissection of pt #12 showing two lumbar postganglionic sympathetic nerve fibres between inferior vena cava and aorta. IVC inferior vena cava

From 1993 through 2017, a total of 722 patients with testicular GCT were treated at Albertinen-Krankenhaus, Hamburg. We elected the cohort treated from 2008 to 2017 (*n* = 378) for review because the EGCCCG guide-lines with the changing role of RPLND came into use in 2008 [13]. Histologies and stage distribution of that cohort are shown in Fig. 1. All patients were managed in line with contemporary guide-lines. Histological work-up of orchiectomy specimens was accomplished according to pathological guide-lines [13]. Clinical staging involved tumor marker measurement prior to orchiectomy and re-evaluation five days postoperatively, also abdominal and chest computed tomography scan with application of intravenous and oral contrast material [17]. A total of 75 cases with NS CS1 were identified in the patient cohort. We retrospectively evaluated the treatment strategies applied in these patients and selected the patients who had undergone RPLND for a closer review. The latter cases were tabulated regarding age, percentage of teratoma and vascular invasion in the primary tumour, numbers of lymph nodes surgically removed and numbers of metastatic nodes, and the particular individual reasons for surgery. The surgical approach consisted of open unilateral nerve-sparing lymph node dissection (Fig. 2) in the Indiana technique [18, 19] and was performed by a single surgeon in all cases (KPD). Frozen section examination was not employed. All patients received a postoperative abdominal drain that was usually removed after 3 to 4 days postoperatively. The rationale for this procedure was to monitor lymphatic fluid drainage and to early detect chylous lymphatic leakage. Statistical analysis involved descriptive statistical methods with calculation of proportions and medians with interquartile ranges (IQRs). The study obtained institutional ethical approval (U3/2015 AKH).

## Results

**Fig. 3:**
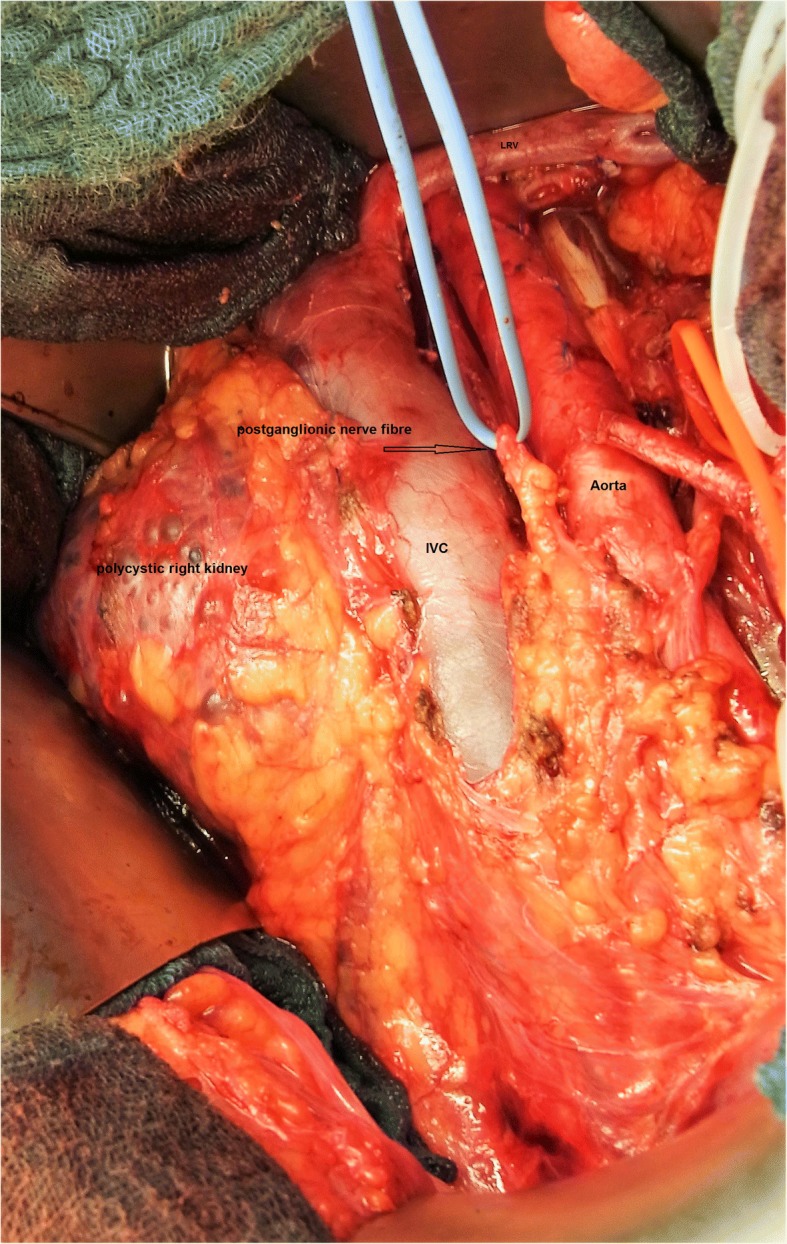
Intraoperative site during RPLND of a patient #5 with polycystic kidney disease. IVC inferior vena cava; LRV left renal vein

**Fig. 4:**
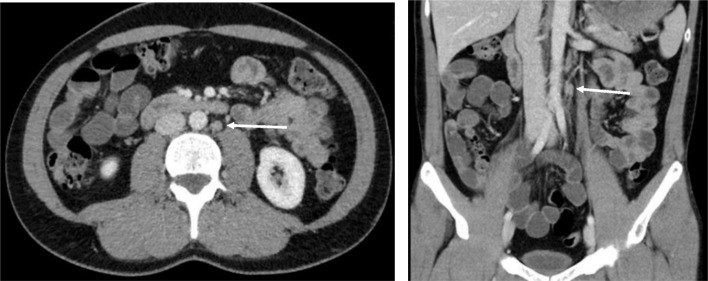
(left) abdominal computed tomography (pt #9) showing lymph node of equivocal size (arrow) in the para-aortal template (axial scan). (right) same patient, CT showing suspicious para-aortal lymph node in coronal scan. Histologically, no metastasis was found in this lymph node

**Fig. 5:**
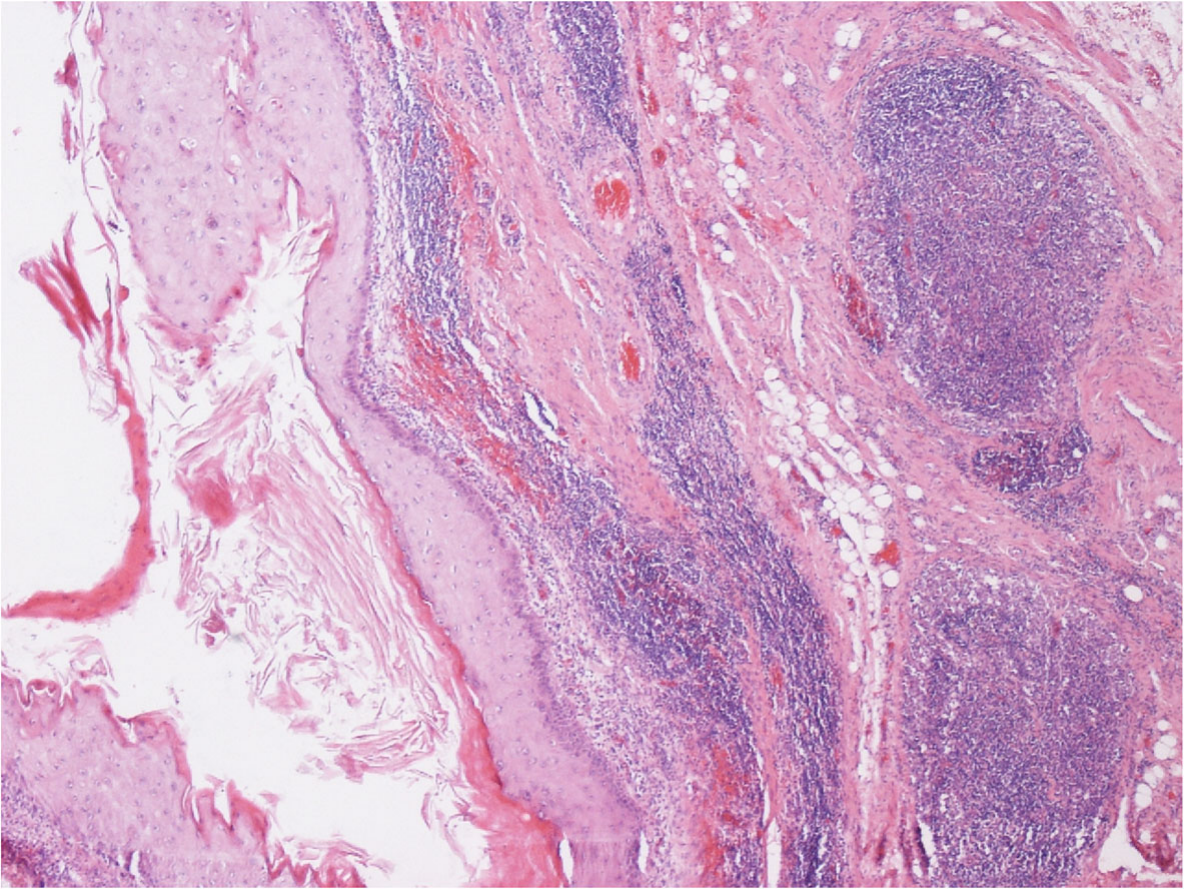
Histologic section of lymph node specimen from RPLND (pt #11). Metastasis consisting of pure teratoma with cystic elements lined by squamous cell epithelium (left side of figure). Intact lymph node tissue on the right side. Hematoxylin eosin stain, original × 100

The treatment strategies applied in the CS1 patients are listed in Table 1. Twelve patients (16%) had received RPLND, clinical details of whom are listed in Table 2. The median ages of surgical patients and those managed with other modalities are 29 years (IQR 25–38, range 18–53 years) and 32 years (IQR 27–40 yrs., range 18–74 years), respectively, and are obviously not dissimilar in view of the widely overlapping interquartile ranges. Regarding histology, all except one patients had primary tumours with components of teratoma, thereof six with proportions of more than 50% teratoma. Among the NS patients managed without RPLND only 35% had components of teratoma in the primary. Vascular invasion was present in 5 of the 12 cases. With regard to tumour markers, alpha fetoprotein was increased prior to orchiectomy in three cases and beta chorionic gonadotropin in one. All patients were marker-negative at the time of decision-making for additional treatment. The particular reasons for electing RPLND instead of chemotherapy or surveillance were unsuitability of chemotherapy due to chronic illnesses in 2 cases (Fig. 3), patient’s choice in 4 cases, and equivocal radiological findings in the presence of teratomatous primary tumour in the remaining six cases (Fig. 4). A median number of 27 (range 10–42) lymph nodes were excised upon surgery. Lymph node metastases were identified in 5 cases (Fig. 5) none of whom had extranodal extension and all were excised completely. Two of the five pN1 patients received adjuvant chemotherapy. The reasons for additional treatment were an apparently high risk of recurrence in the patient with 5 nodes involved (21 yrs., #1, Table 2), and the individual wish for highest probability of disease-free survival in the other one (29 yrs., #12, Table 2).

**Table 1:** Treatment modalities applied after orchiectomy in 75 patients with nonseminomatous testicular germ cell tumours clinical stage 1

	(Number)	(Percent)
Adjuvant chemotherapy ^a^	54	72.0
Surveillance	9	12.0
RPLND	12	16.0

^a^Chemotherapy consisted of two courses of PEB in 35 patients and of one course in 18; one had other chemotherapy

**Table 2:** Synopsis of patients undergoing primary RPLND

Patient (#)	Primary tumour: % teratoma	Primary tumour: Vascular inavasion	Individual reason for RPLND	Surgical result: nodes involved/nodes excised (n/n)	Additional treatment	Outcome
1	75%	no	Teratoma plus equivocal radiological finding	5/15	2xPEB	NED 8 yr
2	20%	no	Patient’s choice	1/27	F/U	NED 7 yr
3	40%	no	Lupus erythematodes, chronic glomerulonephritis	0/42	F/U	NED 7 yr
4	60%	yes	Teratoma plus equivocal radiological finding	0/27	F/U	NED 6 yr
5	20%	yes	chronic kidney disease due to congenital polycystic disease	0/22	F/U	NED 5 yr
6	40%	yes	Equivocal radiological findings	0/30	F/U	NED 4 yr
7	50%	no	Patient’s choice	0/26	F/U	NED 4 yr
8	10%	yes	Equivocal radiological findings	1/33	F/U, NHL 1 year later	AWSM 1 yr
9	95%	no	Teratoma plus equivocal radiological finding	0/24	F/U	NED 3 yr
10	60%	no	Patient’s choice	0/39	F/U	NED 3 yr
11	90%	no	Teratoma plus equivocal radiological finding	1/29	F/U	NED 2 yr
12	0	yes	Patient’s choice	1/10	2x PE	NED 1 yr.

PEB chemotherapy with cisplatin, etoposide, bleomycin; *F/U* follow-up, *NHL* Non Hodgkin lymphoma, *NED* no evidence of disease, *AWSM* alive with second malignancy, *yr* years

No major surgical complications were noted in any of the patients and in all of whom antegrade ejaculation was preserved. One grade 2 complication according to the Clavien/Dindo classification involved chylous lymph secretion after restarting of oral nutrition that was amply detectable in the fluid collected via the abdominal drainage tube. This patient required intravenous nutrition for three days until cessation of chylous leakage. All patients remained disease-free with respect to GCT, however, one patient developed malignant Non-Hodgkin lymphoma one year after treatment for GCT and is currently undergoing chemotherapy for that second malignancy. Of the patients managed with surveillance, two relapsed and were salvaged with chemotherapy. No relapse was recorded in those undergoing adjuvant chemotherapy. However, one patient succumbed to treatment-related vascular complications involving mesenterial infarction with bowel gangrene resulting from cisplatin-based chemotherapy.

## Discussion

Retroperitoneal lymph node dissection is clearly no more the standard way of management of CS1 nonseminoma in European countries [20]. But, as shown in the present series, a well selected sub-cohort of patients might well benefit from it. RPLND is particularly useful in cases where chemotherapy is precluded by concurrent health problems. This constellation was given in two of our patients, one of whom had chronic kidney disease due to congenital polycystic disease (Fig. 3) and the other suffered from lupus erythematosus auto-immune disease. The surgical approach employed in these cases not only obviated the need for upfront chemotherapy but in light of high relapse rates upon surveillance it also minimized the need of chemotherapy during follow-up.

Four patients of our series refused chemotherapy for personal reasons and opted for surgery. All of these decisions were made by the patients after full information about advantages and disadvantages of the available treatment modalities representing the expression of patient autonomy as recently advocated by a joint statement of leading European GCT experts [21]. According to that report, a personalized approach to management decisions should be favoured because the over-all cure rates are excellent regardless of the treatment modality employed [14, 22]. Further, patient autonomy is to be strictly respected by care givers, and professionals are not supposed to influence their patients´ decisions. A full definition of patient autonomy is given on the MedicineNet website (www.medicinenet.com).

In six patients the decision for RPLND was based on equivocal radiological findings in the presence of teratomatous elements in the primary tumour. Retroperitoneal lymph node metastases are radiologically defined by nodes sized > 10 mm in diameter and located in the primary landing zone of the testicular tumour [13, 23, 24]. However, as shown in large series of patients undergoing primary RPLND, around 20–25% of patients may harbor metastatic seeds in lymph nodes despite negative radiological findings [25, 26]. Clearly, a lot of clinical uncertainty exists in cases with lymph nodes sized around 1 cm particularly in those with negative markers. Adjuvant chemotherapy may overcome this problem because 1 or 2 cycles of cisplatin-based chemotherapy will usually sterilize micrometastatic spread [10, 27]. But of note, the subgroup of teratoma does not respond to chemotherapy [28]. In patients having a high proportion of teratoma in the primary tumour, such chemotherapy-resistent elements must also be expected in the secondaries [29–31]. This constellation must be particularly considered when equivocally enlarged para-aortal lymph nodes are found upon abdominal imaging. Accordingly, in three of our six patients with these features, metastases were detected in the RPLND specimens. In two of whom, only one microscopic focus was found and surgery was considered sufficient for cure (Fig. 5).

When RPLND lost its role as the standard way of management of CS1 nonseminoma, the reasoning was mainly based on two arguments, the perioperative risk of this major surgical procedure being the leading one [12, 32]. Perioperative morbidity is clearly undisputable, but it is constantly decreasing ever since the employment of nerve-sparing surgical techniques. Furthermore, reduced complication rates result from rising experience of surgeons based on the increasing acceptance of guide-line recommendations to refer GCT patients requiring specific treatment modalities to recognized centres of excellence [33–36]. A further reduction of perioperative morbidity might be achieved with the upcoming implementation of laparoscopic or robotic-assisted surgical techniques [37].

The other argument against RPLND was the expectation of an over-all increased treatment burden in surgical patients relating to additional measures required in those with metastases found upon surgery (i.e. pathological stage [pS] 2a,b) [12, 32]. By comparison, primary adjuvant chemotherapy usually does not necessitate second treatment measures. However, dual treatment (i.e. RPLND plus adjuvant chemotherapy) is actually required only by a minority of patients. Roughly, one third of CS1 patients undergoing primary RPLND will have pS2a,b [26]. But as shown in the classic reports on primary RPLND, about one half of the patients with surgically proven lymph node metastases do not progress and are thus virtually cured with the procedure [5, 33]. Accordingly, this way of management was successfully applied in two of our cases. Cisplatin-based chemotherapy does effectively eradicate microscopic foci of GCT. One course of PEB is sufficient to control CS1 disease [27].Two courses of PE (without bleomycin) have been shown to be safe in the adjuvant setting of pS2a cases [38] which was confirmed in one of our patients. In conclusion, only a small proportion of about 10–15% of the patients undergoing RPLND will need adjuvant chemotherapy as a second treatment modality, and notably, that treatment can be safely shaped to reduced doses with reduced toxicity. The over-all burden of additional therapy of the surgical patients is probably not as extensive as initially believed.

When weighting the arguments for and against the treatment modalities available for CS1 nonseminoma (i.e. chemotherapy, surveillance, RPLND) it should be noted that we are facing increasing knowledge about hazardous late effects of chemotherapy, particularly in light of the long-term exposure to circulating platinum owing to an estimated half-life of as long as 3.7 years [39]. The risk of second malignancies is significantly increased after cisplatin-based chemotherapy depending on cumulative dosages [40]. An excess of haematological malignancies has repeatedly been reported but also increased rates of renal carcinomas, thyroid cancer and soft tissue neoplasms [41–43]. In addition, multiple organ late toxicities have been reported notably a 1.9–3.1 fold risk of cardiovascular diseases including myocardial infarctions and cerebral strokes [44, 45], but also decreased pulmonary function [46] as well as other significant late sequelae of chemotherapy in a variety of organs [47, 48]. Although all of these late toxicities have been documented so far only in cases receiving full course chemotherapy it is not irrational to assume that late toxicities of lesser extent might occur in patients receiving the abbreviated prophylactic regimens. Particularly in view of the young ages of the nonseminoma patients potential late toxicities of systemic therapy must not be ignored.

Limitations of our analysis mainly involve the low number of patients. Also, selection bias cannot be ruled out because of the single-centre setting and the retrospective design of the study.

## Conclusions

In Europe, primary RPLND is clearly not the standard way of managing nonseminoma CS1 patients. However, as documented herein it can be a valuable option in well-selected patients, particularly those with concurrent chronic diseases. Also, patients with equivocal radiological findings upon abdominal imaging in the presence of teratoma in the primary might benefit from surgery. Finally, a few patients may prefer RPLND after full information about the treatment modalities mirroring the increasing awareness and acceptance of patient autonomy.

## Abbreviations

CI: Confidence intervals
CS: Clinical stage
GCT: Germ cell tumour
IQR: Interquartile range
NS: Nonseminoma
PEB: Chemotherapy with cisplatin, etoposide, bleomycin
pS: Pathological stage
RPLND: Retroperitoneal lymph node dissection
